# Prognostic potential of cardiac structural and functional parameters and N-terminal propeptide of type III procollagen in predicting cardiac fibrosis one year after myocardial infarction with preserved left ventricular ejection fraction

**DOI:** 10.18632/aging.202495

**Published:** 2021-01-11

**Authors:** Anastasia Osokina, Viktoria Karetnikova, Olga Polikutina, Anna Ivanova, Olga Gruzdeva, Yulia Dyleva, Aleksandr Kokov, Natalia Brel, Tamara Pecherina, Olga Barbarash

**Affiliations:** 1 Federal State Budgetary Institution, Research Institute for Complex Issues of Cardiovascular Diseases, Kemerovo 650002, Russian Federation

**Keywords:** myocardial infarction, diastolic dysfunction, heart failure, cardiofibrosis

## Abstract

The aim of the study were to evaluate the prognostic potential of serum level of N-terminal propeptide procollagen type III (PIIINP) and heart parameters for predicting heart cardiac fibrosis 1 year after ST-segment elevation myocardial infarction (STEMI) with preserved left ventricular ejection fraction (LVEF). 68 patients with STEMI and preserved LVEF with acute heart failure of the I-III degree according to the Killip classification were examined. Echocardiography was performed and PIIINP levels were measured on days 1 and 12, as well as 1 year after STEMI. A year after STEMI, was performed contrast magnetic resonance imaging and patients were assigned into four groups depending on the severity of cardiac fibrosis: cardiac fibrosis 0% (n=49, 57% of 86 patients); ≤5% (n=18, 20.9%); 6-15% (n=10, 11.6%); ≥16% (n=9, 10.5%). Direct correlations between the severity of cardiac fibrosis, PIIINP level and indicators of diastolic function were established. The risk of cardiac fibrosis increases at the level of PIIINP ≥381.4 ng / ml on the 12th day after STEMI with preserved LVEF (p=0.048). Thus, measuring the level of PIIINP in the inpatient period can allow timely identification of patients with a high risk of cardiac fibrosis 1 year after STEMI with preserved LVEF.

## INTRODUCTION

Fibrosis is generally considered a progressive process, in which injured tissues are gradually replaced with connective tissue. In addition to the natural aging process, trauma, infectious and allergic diseases, and radiation injury can cause fibrosis. The heart, similar to any other organ, can be subject to fibrosis. Myocardial fibrosis is a common finding in many forms of cardiovascular diseases [1]. Pronounced structural and functional changes in the ventricles culminate in poor myocardial elasticity and contractility [2] that may result in the development of chronic heart failure (CHF) [3, 4].

Therefore, studies of heart failure (HF) with preserved left ventricular function after myocardial infarction are of particular interest. Myocardial fibrosis is one of the most significant mechanisms of the formation and progression of LV myocardial dysfunction. The diagnostic and prognostic potential of a number of serum biomarkers of myocardial fibrosis has been studied. The most promising ones include procollagen precursors, including N-terminal propeptide of type III procollagen [PIIINP]) [5–7]. However, the specificity of serum biomarkers is not high and biomarker levels are known to also be affected by various pathological conditions (osteoporosis, cancer, connective tissue diseases, etc.). Endomyocardial biopsy is a routine method for the diagnosis of myocardial fibrosis. Since this procedure is an invasive one, it is still associated with several complications at a rate of up to 0.8%. Thus, it is important to establish highly informative non-invasive visualizing methods for determining the qualitative and quantitative parameters of fibrosis [8]. In recent years, contrast magnetic resonance imaging (MRI) has emerged as a promising tool to diagnose and evaluate cardiac fibrosis. However, the question regarding the best method to predict the development of fibrosis remains unanswered, since there are no convincing data on the prognostic value of the available biochemical markers of fibrosis, as well as cardiac structural and functional parameters, for the evaluation of patients with myocardial infarction (MI). We hypothesized that echocardiographic indicators with serum biomarkers for fibrosis, evaluated within the in-hospital period after MI, may have beneficial potential for predicting the development of cardiac fibrosis. Our study aimed to evaluate the role of the serum marker for fibrosis—PIIINP—and cardiac structural and functional parameters in the prediction of cardiac fibrosis 1 year after ST-segment elevation myocardial infarction (STEMI) with preserved left ventricular ejection fraction (LVEF).

## RESULTS

### The clinical and demographic data of patients and therapy

The clinical and demographic data of patients enrolled in this study are presented in Table 1. The average age of patients was 57.8 (± 5) years. The vast majority of patients had signs of acute HF corresponding to Killip classes I and II (84.9% and 10.5%, respectively). Four patients (4.6%) had Killip class III HF. There was a high prevalence of cardiovascular risk factors in the study sample. Almost 50% of all patients were active smokers at admission. More than half of them suffered from arterial hypertension (AH), 22.1% of patients had hypercholesterolemia, 30.2% were obese, and 5.8% had a positive history of type 2 diabetes mellitus.

**Table 1:** Clinical and demographic data of the study population (n=86, 100%).

	**n**	**%**
Males	63	73.3
Females	23	26.7
Arterial hypertension	67	77.9
Hypercholesterolemia	19	22.1
Diabetes	8	9.3
Obesity (BMI ≥30 kg/m^2^ according to the WHO classification)	26	30.2
Smoking	47	54.7
Chronic kidney disease	2	2.3
Clinical history of chronic heart failure	6	7.0
Percutaneous coronary intervention (not earlier than 1 year before the present study)	3	3.5

BMI, body mass index; WHO, World Health Organization.

Seventy-nine patients (91.9%) had a SYNTAX score of ≤22. Intermediate and severe coronary artery disease (SYNTAX ≥23) was found in seven patients (8.1%). Sixty-six patients (76.7%) underwent stent implantation, and of those, 25 (29%) received a drug-eluting stent (DES). A residual SYNTAX score of <8 was found in 63 patients (73.2%). Thirteen patients (15.1%) were referred to the next stage for revascularization.

During the in-hospital period, all patients received standard drug therapy according to the European Society of Cardiology guidelines (2015), including heparin, clopidogrel, aspirin, angiotensin-converting enzyme inhibitors (ACE inhibitors), β-blockers, statins, Ca^2+^ channel blockers, and nitrates. At the 1-year follow-up, we found that 71% of patients took antiplatelet agents (27% took dual antiplatelet therapy), 70.1% took ACE inhibitors, 80.3% took β-blockers, 67% took calcium antagonists, 45% took statins, and 19% took nitrates.

At the 1-year follow-up, patients were assigned to four groups based on the MRI findings: Group 1 (n=49, 57%), patients without cardiac fibrosis, whose average age was 57.9 years; Group 2 (n=18, 21%), patients with cardiac fibrosis covering ≤5% of the myocardium, whose average age was 61.2 years; Group 3 (n=10, 12%), patients with cardiac fibrosis ranging between 6% and 15%, whose average age was 55.9 years; and Group 4 (n=9, 10%), patients with cardiac fibrosis over 16%, whose average age was 54.8 years [9]. Patient groups, divided by the severity of cardiofibrosis, were comparable in terms of therapy.

### The echocardiography results

The echocardiography results are presented in Table 2. At the 1-year follow-up, the mean LVEF had significantly decreased compared to day 1 after MI (p=0.018). Fifteen (17.4%) patients reported intermediate LVEF values with a mean value of 53% [47; 56]. e’ started decreasing from day 1 up to the end of the follow-up. The E/e’ ratio showed an increase within 1 year post-MI, indicating the progression of diastolic dysfunction. E_m_ decreased from normal to pathologically low 1 year after STEMI. The obtained values at the three selected time points were significantly different. An increase in the left atrium volume was noted within the 1-year post-MI period. At day 1 post-MI, 25 patients (29.1%) had signs of diastolic dysfunction. However, the number had increased by 10.5% (n=9) by the end of the follow-up.

**Table 2:** Progress in echocardiography parameters within 1 year after STEMI.

**Parameters**	**Selected time points**				**p**
	**control group**	**1st day**	**12th day**	**1 year**	
LVEF (%)	66.0 [63.0; 69.0]	59.0 [54.0; 63.0]	62.0 [56.0; 65.0] ^* #^	53.0 [47.0; 56.0] ^*^	<0.001
E_m_ (cm/s)	8.9 [8.3; 9.1]	7.2 [6.3; 7.8]	6.4 [4.2; 7.9] ^#^	6.5 [4.0; 7.3] ^*^	0.048
E/ e’	7.1 [6.8; 7.7]	9.9 [9.4; 10.2]	11.1 [9.3; 13.2] ^* #^	13.9 [12.1; 14.5] ^*^	0.027
mPAP, mmHg	15.0 [14.0; 21.0]	25.0 [21.0; 26.0]	25.0 [23.0; 27.0]	24.0 [21.0; 28.0]	0.157
e’ (cm/s)	8.8 [8.1; 9.2]	9.0 [8.6; 11.4]	8.8 [7.5; 10.4] ^#^	8.6 [7.2; 9.4]	0.047
LA volume, mL	54.8 [51.5; 58,4]	80.0 [73.0; 90.0]	84.0 [77.0; 92.0] ^*^	84.5 [79.0; 95.0] ^*^	0.004

Note: * p<0.05 in comparison with the time point A, # p<0.05 in comparison with the timepoint C.

STEMI, ST-segment elevation myocardial infarction; LVEF, left ventricular ejection fraction; mPAP, mean pulmonary artery pressure; LA, left atrium; Em (cm/s)- displacement of the lateral part; e’ (cm/s) – e’- displacement of the septal part; LA volume, mL – volume of the left auricle.

### Progress in PIIINP levels within 1 year after STEMI

**Figure 1:**
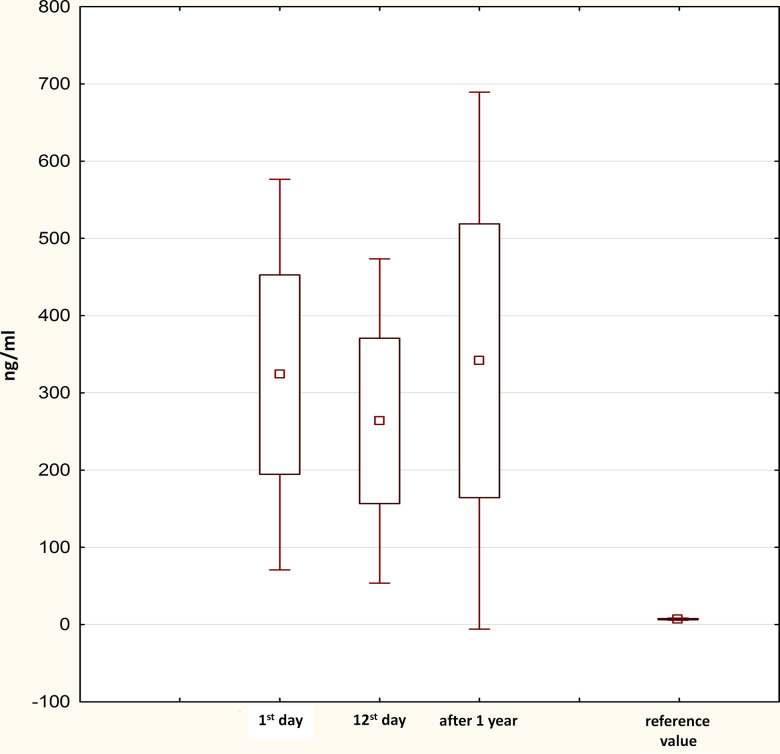
**Progress in PIIINP levels within 1 year after STEMI.** PIIINP, N-terminal propeptide of Type III procollagen; STEMI, ST-segment elevation myocardial infarction.

Figure 1 shows the progression of PIIINP concentrations. Elevated levels of PIIINP at day 1 post-MI (311.2 [220.1; 376.3] ng/mL) decreased to 223.3 [195.3; 312.1] ng/mL at day 12 post-MI and then reached the initial values 1 year after STEMI (312.6 [228.0; 383.8] ng/mL). There were significant differences between the A and B (p<0.001) and B and C (p=0.002) time points.

### The changes in LVEF in the study groups within 1 year post-MI

**Figure 2:**
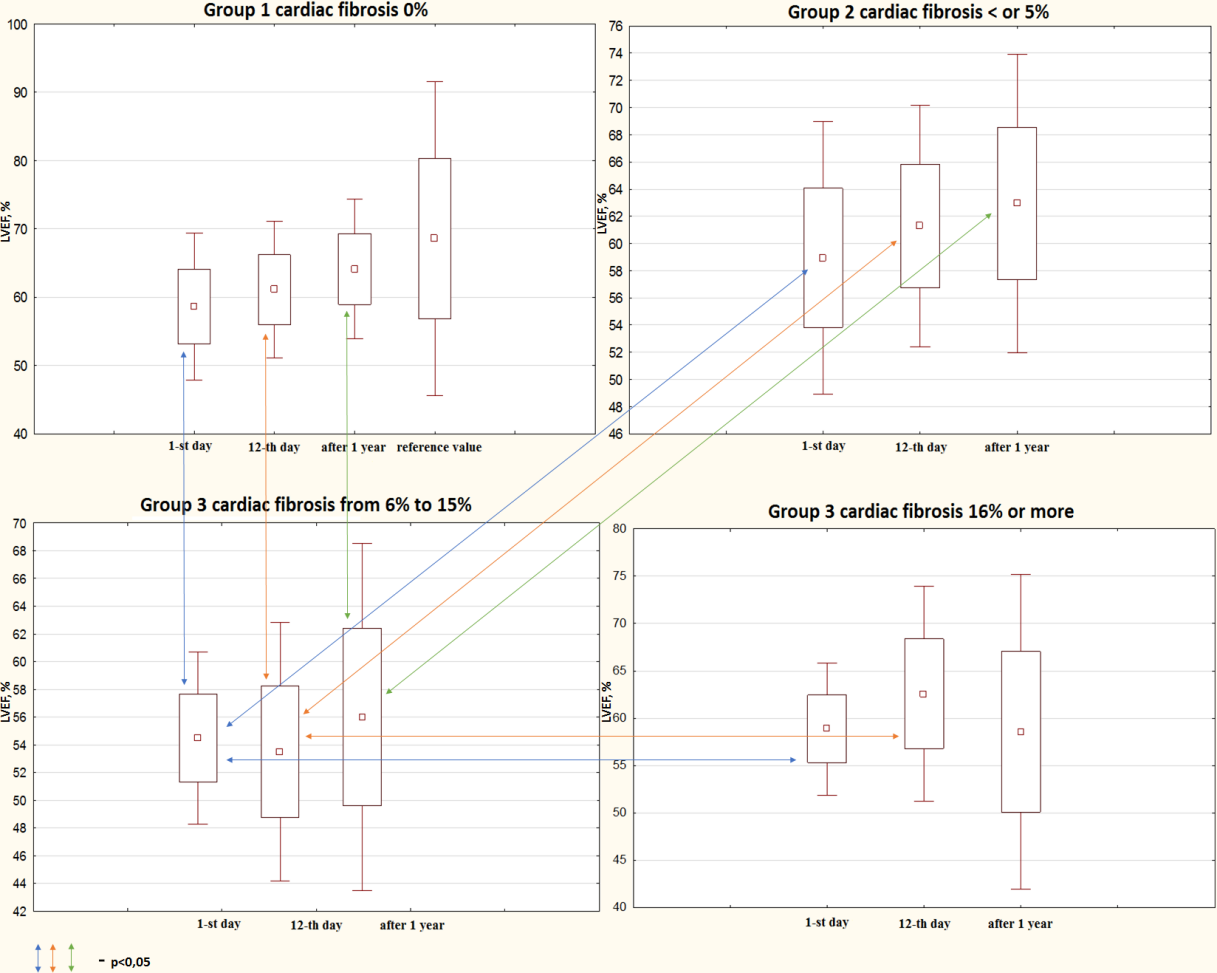
**Progress in systolic function depending on cardiac fibrosis according to the MRI findings within the 1-year follow-up.** MRI, magnetic resonance imaging; LVEF, left ventricular ejection fraction.

In addition to the evaluation of the progress in echocardiographic parameters and PIIINP levels in the total sample, we focused on a more detailed analysis of these parameters in relation to the severity of cardiac fibrosis according to the MRI findings. Figure 2 shows the changes in LVEF in the study groups within 1 year post-MI. Patients without any signs of cardiac fibrosis demonstrated a significant improvement in LVEF from one selected time point to the other. LVEF increased from 60% [54; 63] at day 1 post-MI to 62% [60; 65] at discharge (p<0.001). At the 1-year follow-up, LVEF reached 64% (62; 68) (1st day vs. 1-year, p<0.001; 12th day vs. 1-year, p=0.038).

Patients with cardiac fibrosis covering up to 5% of the myocardium demonstrated a significant improvement in LVEF only in the in-hospital period, when LVEF increased from 60% [56; 63] to 63% [60; 64]% (p=0.038). Despite further improvement up to 65% [61; 65], changes were statistically insignificant. Another trend was determined in patients with cardiac fibrosis ranging from 6% to 15%. Contractile function decreased in this group at each control time point.

Initial LVEF 55.5% [51; 57] decreased to 54% at discharge and did not reach the initial value by the end of the follow-up (54.5% [53; 62]). However, the negative trend in LVEF changes was statistically insignificant. Interestingly, patients with the highest fibrotic area demonstrated a rather specific trend that was different from the others. LVEF increased from 60% [57; 61.5] at day 1 post-MI up to 65% [62; 66] at discharge and then decreased to the initial value of 61% [50; 64] 1 year after MI. The abovementioned changes did not reach statistical significance.

At day 1, patients from Group 1 and 2 had a similar mean LVEF (60%) that was significantly higher than that in Group 3 (55.5%). Moreover, patients from Group 3 and 4 also demonstrated similar mean LVEFs that corresponded to the mean LVEF in Group 1 and 2 (60%) (Group 3 vs. Group 4, p=0.015). At time point B, patients with cardiac fibrosis covering 6% to 15% of the myocardium had a lower mean LVEF (54%) than that in Group 1 (62%), Group 2 (63%), and Group 4 (65%). At the 1-year follow-up, patients from Group 1 and 2 demonstrated a higher mean LVEF (64% and 65%, respectively) than that in Group 3 (54.5%).

### The progress in diastolic dysfunction in the study groups within 1 year after STEMI

At day 1 post-MI, 25 patients (29.1%) had diastolic dysfunction. This number increased by nine patients (10.5%) by the end of the follow-up. The changes in diastolic function were estimated depending on the severity of cardiac fibrosis 1 year after MI (Table 3). The number of patients with diastolic dysfunction increased in all three groups with cardiac fibrosis, but the proportion varied. Importantly, there were no Group 1 patients with impaired diastolic function, both at day 1 post-MI and 1-year post-MI.

**Table 3:** Progress in the diastolic dysfunction according to the echocardiography findings during the follow-up.

**selected time point**	**Group 1, n**	**Group 2, n**	**Group 3, n**	**Group 4, n**
1 day post-MI	0	1	10	14
1 year post-MI	0	2	12	21

MI, myocardial infarction.

### The progress in PIIINP levels in the study groups in relation to the severity of cardiac fibrosis

**Figure 3:**
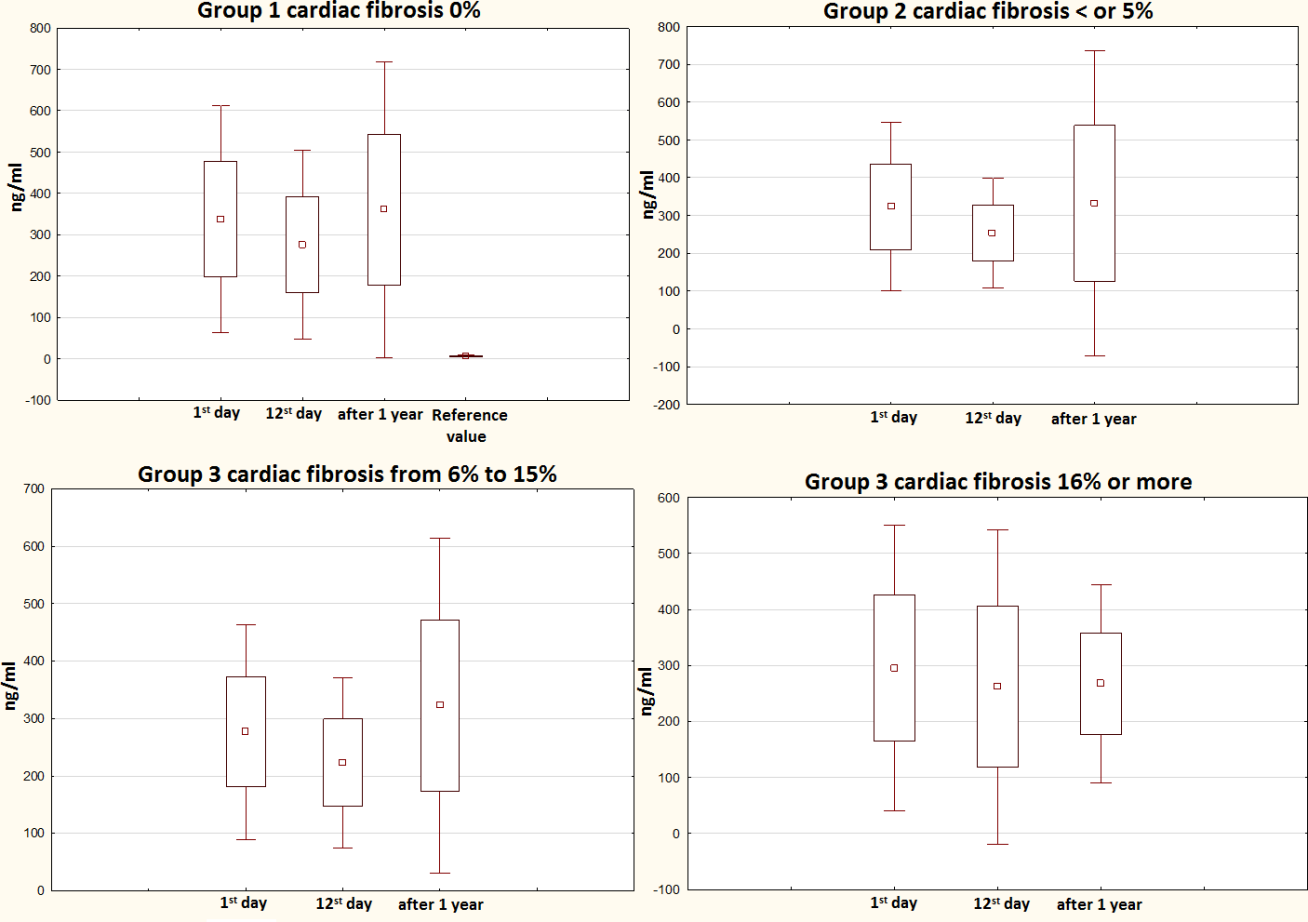
**1-year progress in PIIINP levels in relation to the severity of cardiac fibrosis evaluated by MRI.** PIIINP, N-terminal propeptide of Type III procollagen; MRI, magnetic resonance imaging.

Figure 3 shows the changes in PIIINP levels in the four study groups depending on the degree of cardiac fibrosis. The between-group comparison at each time point did not show any significant differences. Nevertheless, significant differences were found when comparing each group at different study time points. Patients without cardiac fibrosis showed differences between the in-hospital period (p<0.05) and day 12 post-MI and 1-year post-MI (p <0.05), whereas patients with a 5% fibrotic area reported differences only showed differences for the in-hospital period (p<0.05).

### Correlation analysis

**Figure 4:**
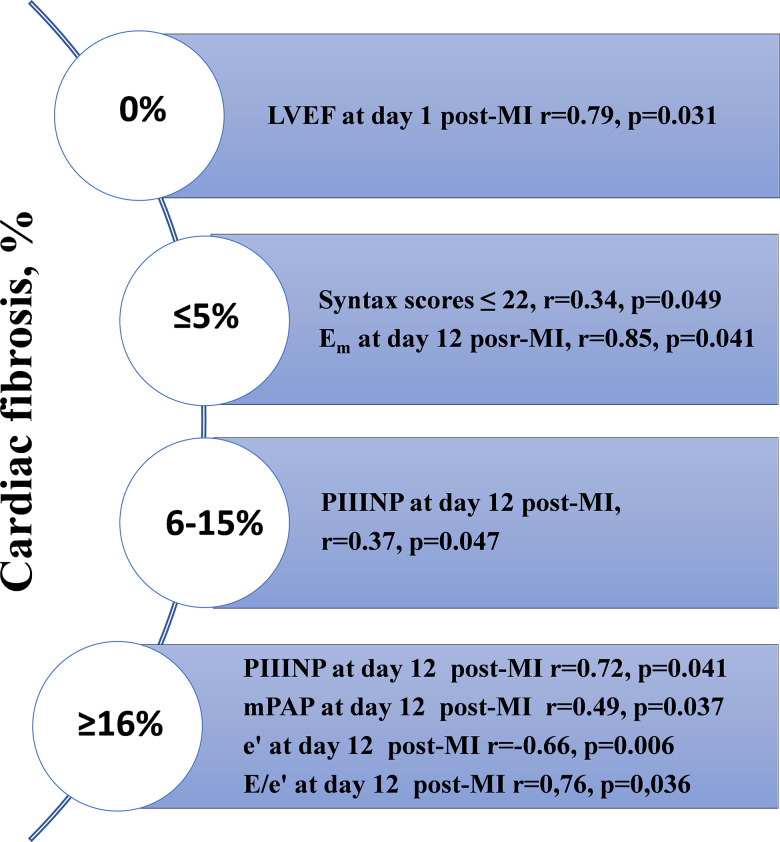
**The results of the correlation analysis depending on the prevalence of cardiac fibrosis measured with MRI one year after STEMI with preserved LVEF.** MRI, magnetic resonance imaging; LVEF, left ventricular ejection fraction; STEMI, ST-segment elevation myocardial infarction; MI, myocardial infarction; mPAP, mean pulmonary artery pressure.

The relationships between the degree of cardiac fibrosis and indicators of diastolic function (echocardiography), serum levels of PIIINP, and the clinical and medical history of patients are presented in Figure 4.

A direct correlation between the severity of cardiac fibrosis, serum PIIINP levels, and diastolic function was found. Serial evaluation of diastolic function parameters showed a negative trend in e’, indicating the aggravation of diastolic dysfunction within 1 year post-MI. It is worth noting that the highest degree of cardiac fibrosis (≥16%) was associated with higher concentrations of PIIINP at day 12 post-MI. The direct correlations between LVEF at day 1 post-MI and the absence of cardiac fibrosis 1 year after STEMI, mean pulmonary artery pressure (mPAP) at day 12 post-MI, and severe cardiac fibrosis (≥16%) were found. Most parameters suggesting diastolic dysfunction at day 12 post-MI (e’, mPAP, E/e’) showed reliable relationships with severe cardiac fibrosis.

### ROC analysis

**Figure 5:**
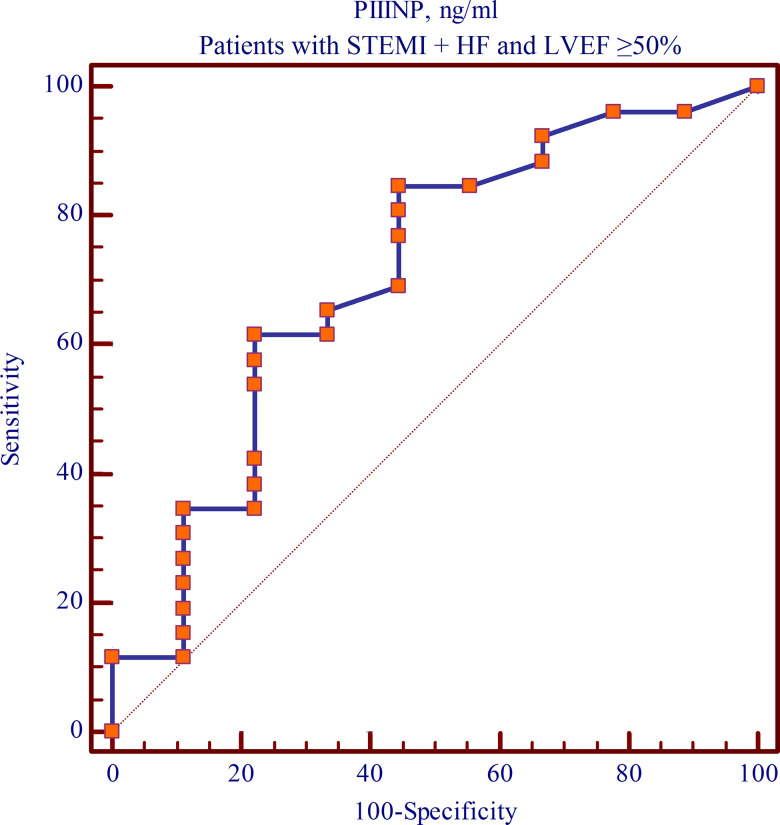
**The cut-off value of PIIINP level at day 12 post-MI with preserved LVEF within 1-year follow-up (ROC curve).**

Receiver operating characteristic (ROC) analysis in Group 4 patients enabled the prognostic value of PIIINP in predicting the development of cardiac fibrosis 1 year after myocardial infarction to be determined. While generating the ROC curve, the PIIINP cut-off level was selected using the stepwise approach to achieve the total maximum sensitivity and specificity of the model. The resultant ROC curve showed that the PIIINP level at day 12 post-MI was associated with the development of cardiac fibrosis within 1 year after the indexed event. Thus, the risk of developing cardiac fibrosis 1 year after MI increased (p=0.048, sensitivity of 84.62%, specificity of 55.56%) with a PIIINP level of ≥381.4 ng/mL at day 12 post-MI with preserved LVEF (Figure 5).

## DISCUSSION

Our study demonstrated that 43% of patients had signs of cardiac fibrosis of varying severities according to the MRI findings 1 year after STEMI with preserved LVEF. Almost half of all patients with cardiac fibrosis (n=19, 22.1%) had the unfavorable result of a fibrotic area of ≥16% [8]. Myocardial fibrosis is considered to be a complex process involving all cellular components of the myocardium [10]. In addition, fibrosis may violate cardiomyocyte supply and impede the electrical contact between them, exacerbate hypoxia due to a decreased density of capillaries, and lead to a deterioration in metabolic processes and ventricular function. All these processes contribute to the changes in the wall thickness and ventricle sizes, as well as systolic and diastolic alterations. The development of diastolic dysfunction within this period subsequently leads to the development of diastolic HF [11].

The adverse course of the long-term post-infarction period in the study population was confirmed by an increase in the number of patients with signs of diastolic dysfunction according to the echocardiography findings. It should be emphasized that diastolic dysfunction is not necessarily a criterion for HF [12], but it is commonly considered an unfavorable prognostic marker in patients with MI. The medical literature presents the evolution of the methods and approaches for a more accurate estimation of diastolic function in routine clinical practice. An increase in the number of studies focused on the evaluation of left ventricular diastolic dysfunction is driven by a high prevalence of diastolic HF among patients with coronary artery disease and the adverse course of a long-term post-infarction period [13].

In addition to instrumental methods for the diagnosis of cardiac fibrosis and diastolic dysfunction, an unfavorable trend in serum PIIINP levels was found. Matrix metalloproteinases-1, -3, -8, -9 (MMP-1, -3, -8, -9), a tissue inhibitor of matrix metalloproteinases-1, -4 (TIMP-1, -4), connective tissue growth factor, C-terminal telopeptide of type I collagen (CTX), galectin-3, and stimulating growth factor (ST2) are the most commonly studied markers [5–7]. In our study, we focused on the changes in PIIINP levels, one of the markers of collagen synthesis. Procollagen precursors containing collagen I carboxy-terminal propeptide (PICP) and PIIINP are the well-known sources of synthesis of type I and III collagen. The structure of cardiomyocytes and the alignment of myofibrils in them is ensured by type I and III collagen. Changes in collagen synthesis and degradation underlay the process of cardiac remodeling. The predominance of synthesis of type III collagen over synthesis of type I collagen leads to myocardial fibrosis and the loss of connection between cardiomyocytes. All these processes result in damage to the structure and the direct function of the myocardium [14]. Elevated PIIINP levels at day 1 post-MI represent a natural response to a vascular catastrophe, accompanied by the activation of the sympathoadrenal system, inflammation, and fibrogenesis. We may assume that the expression of fibrotic markers during the in-hospital period initiates the development of cardiac fibrosis and accelerates its progression during the long-term period. The reported associations suggest the high prognostic potential of serum PIIINP in predicting the development of cardiac fibrosis 1 year after MI in patients with preserved systolic function. An increase in the number of patients with diastolic dysfunction at the 1-year follow-up indicates the progression of HF, which is not yet characterized by clear clinical manifestations.

Correlation analysis of echocardiography parameters and the presence of cardiac fibrosis has provided new data for the biomedical society. The positive relationship between severe cardiac fibrosis and an area of ≥16% with diastolic function at day 12 post-MI was established. A decrease in e’, mPAP, and E/e’ from the day 1 post-MI measurements should be regarded as an alarm in terms of predicting cardiac fibrosis in the long-term period after MI with preserved LVEF [10].

The favorable course of the early post-infarction period in patients with preserved LVEF has been previously shown to be counteracted by a high rate of recurrent MI, decompensated HF, and death in the long-term period [1]. Therefore, it is crucial to detect patients at high risk of these complications as early as possible. The use of routine echocardiography allows indirect assessment of the severity of myocardial fibrosis.

It is worth remembering that MRI can detect already developed cardiac fibrosis of varying severities [15]. This method does not facilitate prediction of the development of cardiac fibrosis, diastolic dysfunction, or diastolic HF in the long-term period. Therefore, assessment of procollagen propeptides seems to be a promising solution to this problem. Our study results indicate the possibility of predicting cardiac fibrosis in the long-term period after MI by determining the concentration of serum PIIINP at day 1 post-MI and day 12 post-MI. The diagnostic approach proposed in our study may allow detection of patients at high risk of cardiac fibrosis and subsequent diastolic dysfunction and progressive deterioration of myocardial function.

Our study has demonstrated a worsening of left ventricular systolic and diastolic function during the year after STEMI with preserved LVEF. Statistically significant correlations between the most unfavorable fibrotic area and echocardiographic parameters of diastolic function and serum PIIINP levels at day 12 post-MI and 1-year post-MI were revealed. Measurement of PIIINP levels in the in-hospital period (day 1 post-MI and day 12 post-MI) is reasonable as it may allow timely identification of patients at a high risk of developing cardiac fibrosis 1 year after STEMI with preserved LVEF.

## MATERIALS AND METHODS

### Protocol approval

The study protocol was approved by the Local Ethics Committee of the Research Institute for Complex Issues of Cardiovascular Diseases. Patients were recruited according to the inclusion and exclusion criteria (see below) over a period of 46 days in 2015. Recruitment was performed in accordance with the guidelines for Good Clinical Practice and the principles of the Helsinki Declaration. All patients provided written informed consent before their participation.

### Study participants

The inclusion criteria were as follows:

- STEMI diagnosed according to the European Society of Cardiology guidelines (2015)
- age ≥18 years
- written informed consent
- acute HF of Killip classes I–III
- LVEF ≥50% (at day 1 post-MI)

The exclusion criteria were as follows:

- acute coronary syndrome as a complication of percutaneous coronary intervention or coronary artery bypass grafting
- acute HF of Killip class IV
- clinically significant concomitant pathology (exacerbation of chronic diseases, the presence of mental disorders)
- prior MI
- LVEF <50% (at day 1 of STEMI)
- death in the first days of hospitalization

### Study population

A total of 86 patients with STEMI and preserved LVEF were included in the study. The mean age of patients was 57.8 (± 5) years. There were more men in the study population (n=63 [73.3%]). There were 23 postmenopausal women (26.7%). All patients underwent standard laboratory and instrumental examinations in order to verify the diagnosis of MI. In addition, all patients underwent coronary angiography using an INNOVA 3100 angiographic system (General Electric, Milwaukee, MI, USA), followed by the stenting of a symptom-dependent artery.

The control group comprised 20 age- and sex-matched healthy volunteers (57.9 years; 75% male [n=15] and 25% female [n=5]).

### Echocardiography

Echocardiography was performed at day 1, day 12, and 1 year after STEMI using a Sonos 2500 device (Hewlett Packard, Andover, MA, USA) [9]. The standard set of parameters was evaluated, including left ventricular global systolic function, left ventricular wall thickness, generally accepted dimension and volume indicators, the presence and the size of the area of dyskinesia in the necrosis and scarring zone, function of the valves, aneurysm, papillary muscle rupture, and myocardial rupture. LVDF was interpreted based on the measurement of the transmitral flow in the pulse-wave Doppler mode and displacement of the mitral valve annulus in the tissue Doppler mode (e’-displacement of the septal part and E_m_ — displacement of the lateral part). LVEF fraction was calculated using the Simpson method. Diastolic dysfunction (DD) was confirmed in the presence of the following criteria:

- E_m_ <10 cm/s
- e’ <8 cm/s
- left atrium volume index >34 mL/m^2^

### Laboratory assays

### Enzyme-linked immunosorbent assay (ELISA)

Serum PIIINP levels were measured using an ELISA with laboratory kits (Cloud-Clone Corp., Katy, TX, 77494, USA) at day 1 post-MI, at day 12 post-MI and 1 year after STEMI in all patients. Serum PIIINP levels in the study sample were compared with the values measured in the control group. PIINP concentration in the control group was 7.2 [6.8; 7.5] ng/mL.

### Assessment of cardiac fibrosis

One year after MI, cardiac fibrosis was assessed by determining the percentage of the myocardial scarring. Patients underwent cardiac MRI using an ExelartAtlas 1.5-T MRI scanner (Toshiba, Tokyo, Japan). Gadolinium-based contrast agent was delivered with a concentration of 0.5 mmol/mL during MRI. In order to visualize the zones with cardiac fibrosis (myocardial zones with delayed gadolinium enhancement after the contrast agent injection), image acquisition was performed 6 minutes after gadolinium contrast administration with a T1 weighted spin echo pulse sequence (TE = 24 ms, TR = 1000 ms, FA = 90°, matrix = 256×256, slice thickness = 7 mm) by slicing along the short axis of the left ventricle. The obtained DICOM images were processed and analyzed using the free software Segment version 2.0 R 4265 (Medviso AB, Lund, Sweden). The percentage of cardiac fibrosis of the total mass of the myocardium was automatically calculated.

### Statistical analyses

Statistical analyses were performed using commercially available Statistica 7.0 software package (StatSoft software package, Tulsa, OK, USA). The data were not normally distributed. Therefore, nonparametric methods were used. The data are presented as median and interquartile range (Me [25per; 75per]). Two independent groups were compared using the Mann-Whitney U test. Three independent groups were compared using the Kruskal-Wallis test by ranks, followed by a pairwise comparison using the nonparametric Mann-Whitney test with the Bonferroni correction. The comparison of dependent groups was performed using the Wilcoxon test. The dependence between the variables was studied with the Spearman rank correlation coefficient. The relationship between several independent variables was determined using a ROC curve. A p value of less than 0.05 was considered statistically significant.
